# Analysis of the antimicrobial mechanism of porcine beta defensin 2 against *E*. *coli* by electron microscopy and differentially expressed genes

**DOI:** 10.1038/s41598-018-32822-3

**Published:** 2018-10-02

**Authors:** Rui-bo Chen, Kun Zhang, Heng Zhang, Chun-yu Gao, Chun-li Li

**Affiliations:** Department of Animal and Veterinary Science, Henan Agricultural University, Zhengzhou, 450002 Henan The People’s Republic of China

## Abstract

Porcine beta defensin 2 (pBD2) is a cationic antimicrobial peptide with broad spectrum antibacterial activity, which makes it a potential alternative to antibiotics to prevent and cure diseases of pigs. However, development of pBD2 as an effective antibiotic agent requires molecular understanding of its functional mechanism against pathogens. In this study, we investigated the functional mechanism of pBD2 antibacterial activity. *Escherichia coli* was incubated with different pBD2 concentrations for different times. Electron microscopy was used to analyze the locations of pBD2 and its induced morphological changes in *E*. *coli*. Gene expression analysis was also performed to further understand the molecular changes of *E*. *coli* in response to pBD2 incubation. The results demonstrated that *E*. *coli* membranes were broken, holed, and wrinkled after treatment with pBD2, and pBD2 was located on the cell membranes and manly in the cytoplasm. Furthermore, 38 differentially expressed genes (DEGs) were detected, successfully sequenced and confirmed by quantitative real-time PCR (qRT-PCR). Most of the known functional DEGs were associated with DNA transcription and translation and located in the cytoplasm. Collectively, the results suggest that pBD2 could have multiple modes of action and the main mechanism for killing *E*. *coli* might be influence on DNA transcription and translation by targeting intracellular molecules after membrane damage, although transport and metabolism proteins were also affected.

## Introduction

The long-term use and abuse of antibiotics have increased pathogen resistance to antibiotics and produced a serious worldwide health problem in farm animals and humans. Due to the pathogens resistance, lack of effective antibiotic treatments has not only restricted the development of pig industries but also endangered human health^1–3^. To overcome this problem, it is extremely important to continuously discover novel antimicrobial agents and understand their functional mechanism of defense against pathogens. Defensins are a family of low-molecular-weight antimicrobial peptides (AMPs) that are secreted by organisms and can be used as potential alternatives for novel therapeutic drugs due to their broad-spectrum activity against pathogens. In addition, these peptides play important roles in innate and adaptive immunity^4–8^. Mammalian defensins are classed into alpha, beta and theta defensins based on the connectivity of three disulfide bridges of cysteine residues^8^. These proteins consist of positively charged amino acid residues, such as lysine and arginine, which contribute to their higher pI values ranging from +5 to 12 as monomers, which is necessary for their functional activity^7–11^.

Genomic sequence analysis revealed that beta defensins are one of the major classes of defensins that are expressed in pigs, but their functional activity and mechanism are not yet completely understood^12–14^. Porcine beta defensin 2 (pBD2) is one of the beta defensins secreted by pigs and has shown high antibacterial activity against gram-negative and gram-positive bacteria including multi-resistant bacteria. In addition, pBD2 has low hemolytic activity against porcine blood and high salt resistance, which qualifies pBD2 as a good antibiotic candidate^15,16^.

Application of defensins as antibiotic agents requires a clear understanding of their antimicrobial mechanisms. Several studies have focused on the mechanisms of interaction between defensins and cell membrane and revealed that positively charged residues of defensins interact with negatively charged components (lipopolysaccharides or phospholipids) in microbial membranes to disrupt the cell membrane as the first step in killing bacteria^17–19^. However, destruction of the extracellular membrane is not sufficient to cause bacterial cell death, and defensins further bind to protoplast membranes to kill bacteria, as described in several studies^17–19^. Several prominent models (called variously the barrel-stave, carpet, toroidal pore, and aggregate models) have been proposed to explain the interaction between the membrane and peptide^10,20^. Defensins have been characterized as either directly killing the bacteria by membrane destruction and decomposition or leading to cell death by altering the permeability of the cytoplasmic membrane and energy state of the cell, as well as by attacking internal targets, such as negatively charged DNA or RNA^21–29^, which have also been classified as membrane-disruptive and nonmembrane-disruptive mechanisms of peptide antimicrobial activity^10,11,30^.

However, usual mechanisms of defensins have been studied in different organisms, and the antibacterial mechanisms may vary based on the organism, class and type of AMP^30,31^. The mechanisms of beta defensins have not been well studied. In particular, the mechanism of pBDs has not been completely defined and must be investigated to develop novel antimicrobial agents for porcine diseases.

In this study, we investigated the antibacterial mechanisms of pBD2 using electron microscopy and DEGs analysis. *E*. *coli* was incubated with different concentrations of pBD2 for different exposure times. Morphological changes of pathogens were observed by scanning electron microscopy (SEM), and the locations of pBD2 were detected by immunofluorescence microscopy (IFM) and immuno-gold transmission electron microscopy (TEM). Moreover, the DEGs were identified by ACP-based RT-PCR and confirmed by quantitative real-time PCR (qRT-PCR). DEGs were further subjected to functional annotation using BLAST analysis to investigate the molecular mechanisms of pBD2 against *E*. *coli*.

## Results

### Preparation of pBD2

**Figure 1:**
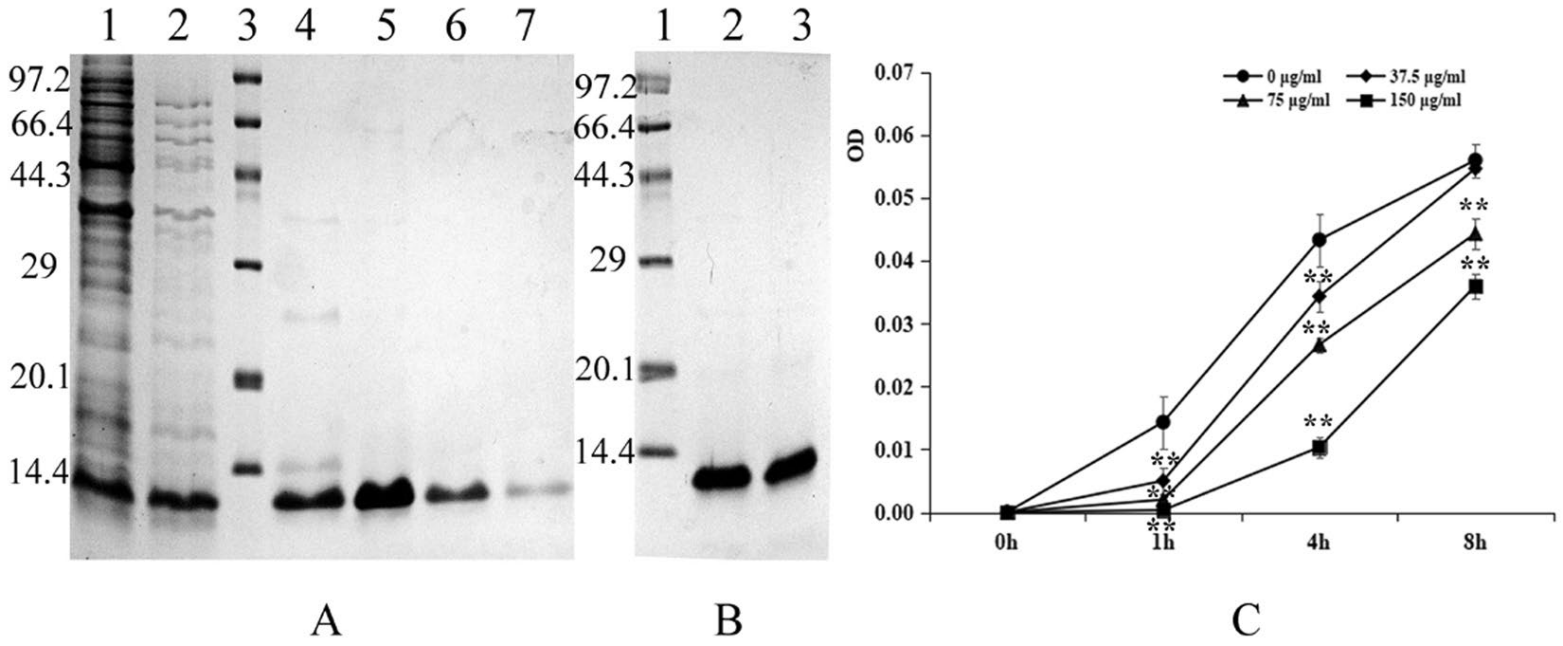
Analysis of expressed fusion and purified pBD2 and its antimicrobial activity. (**A**) Analysis of expressed fusion pBD2. Lane 1 indicates the induced protein; Lane 2 indicates the protein after boiling at 80 °C for 10 min; Lane 3 indicates the protein marker; Lanes 4–7 indicate the 4–7 fractions after purification of pBD2 by HisTrapTMHP chromatography; (**B**) Analysis of the purified protein. Lane 1 indicates the protein marker; Lanes 2–3 indicate the purified fusion protein after enrichment; (**C**) Antimicrobial activities of pBD2 against approximately 10^9^ cfu/mL *E*. *coli* at different time points. OD values at 630 nm (reference 405 nm) were measured at different times at various pBD2 concentrations. The OD value at 0 h was adjusted to 0, and the increased OD values were calculated. The full-length gels are presented in Supplementary Figure 1.

Based on a BL21(DE3)-pET*-pBD2* constructed in our lab, the recombinant pBD2 with a His-tag in the N-terminal was induced and purified as described previously^15^. The molecular weight of the purified recombinant pBD2 was approximately 12 kDa, a value close to its theoretical mass as analyzed by Gel-ProAnalyzer (4.0) (Fig. 1A,B). The results showed that the purified pBD2 had high purity.

### Antibacterial activity

The growth of bacteria was measured by optical density, and the increased OD values were compared with those at 0 h shown in Fig. 1C. The increased OD value was significantly decreased gradually along with the pBD2 concentrations increased at the same time points (*P* < 0.01), but obviously increased along with the exposure time (*P* < 0.01). These results indicated that the recombinant pBD2 had high antimicrobial activity against *E*. *coli* with dose-related effects.

### Scanning electron microscopy

**Figure 2:**
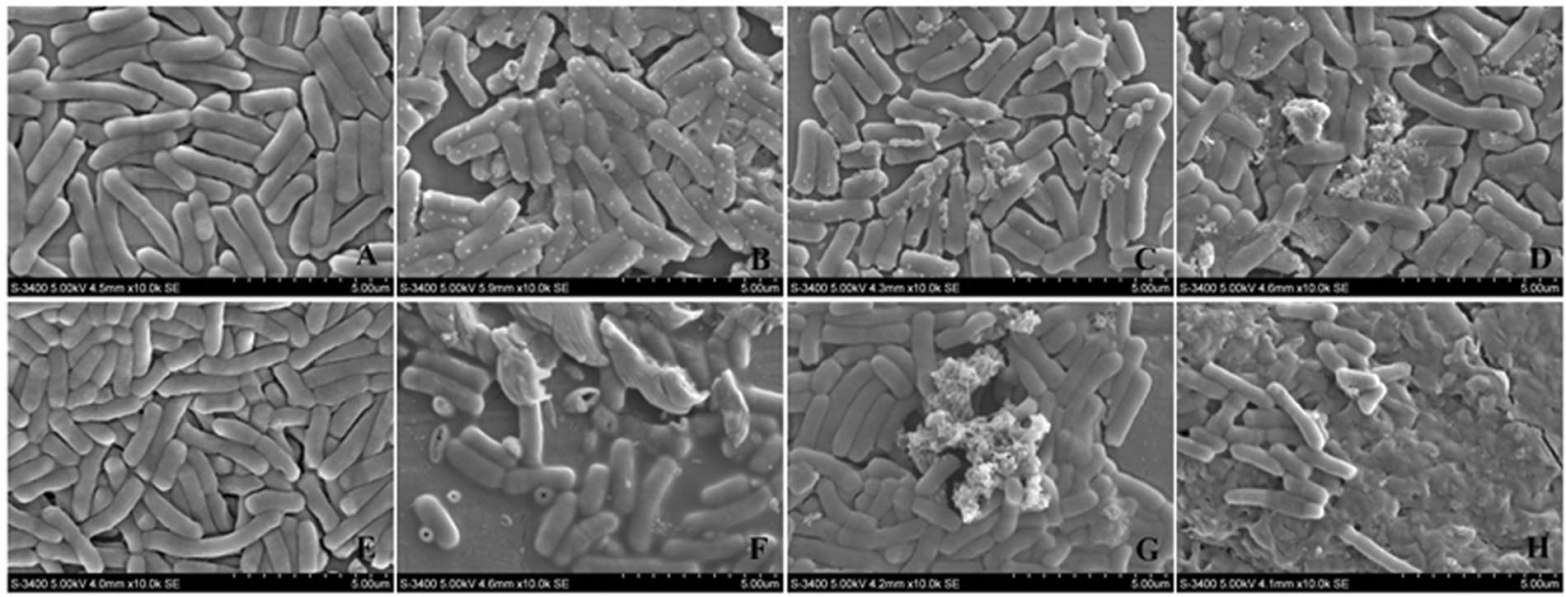
Morphological changes of *E*. *coli* after treatment with fusion protein pBD2 by scanning electron microscopy. (**A**–**D**) Show *E*. *coli* treated with fusion pBD2 for 1 h at 0, 37.5, 75, and 150 μg/ml, respectively; (**E**–**H**) show for 4 h at 0, 37.5, 75, and 150 μg/ml, respectively. The images (a–h) are representative of 96 images observed in three independent experiments.

The *E*. *coli* cells were incubated for 1 h and 4 h with different concentrations of pBD2 (0, 37.5, 75 and 150 μg/mL). Morphological changes were analyzed and are shown in Fig. 2. Bacterial cells from the control group were intact and smooth without noticeable damage (Fig. 2A,E). In contrast, *E*. *coli* cells treated with different concentrations of pBD2 were damaged, as evident by outflow of cell contents (Fig. 2C), deep craters and burst cells (Fig. 2B,F), debris of cells and cell death (Fig. 2D,F–H), and so on. Morphological damage occurring in response to different doses of pBD2 was similar. The damage was more serious and noticeable at 4 h than that at 1 h. Furthermore, the numbers of cells were reduced with increased pBD2 concentrations. The results demonstrated similar morphological changes irrespective of pBD2 concentration, whereas more pronounced effects were observed with increased incubation time.

### Cellular localization of pBD2 peptides

**Figure 3:**
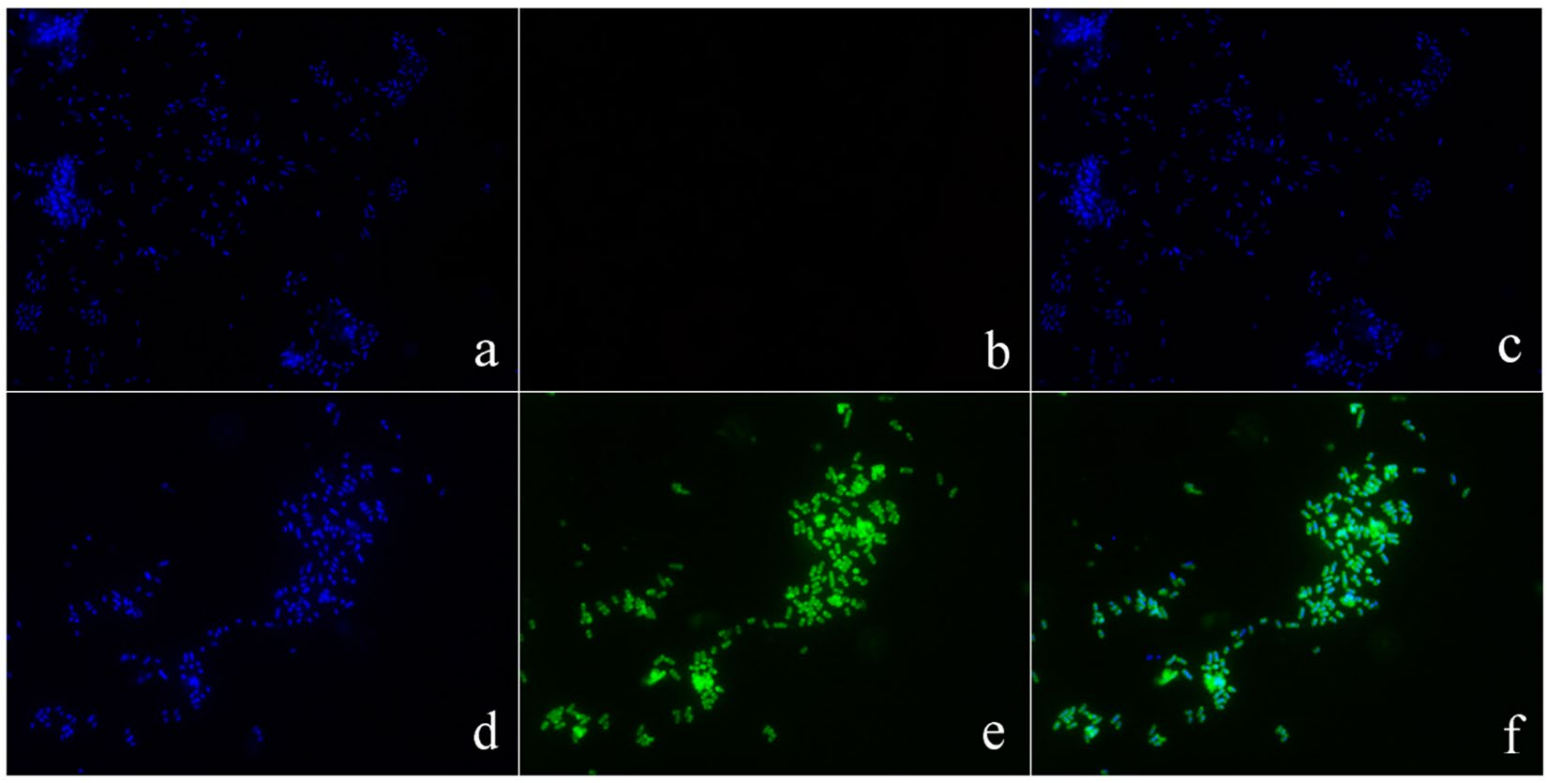
Analysis of *E*. *coli* after treatment with fusion protein pBD2 by immunofluorescence microscopy. (**a**–**c**) Show *E*. *coli* (without first antibody) as the control dyed by DAPI, FITC and DAPI/FITC, respectively; (**d**–**f**) show *E*. *coli* treated with 150 μg/ml pBD2 for 1 h dyed by DAPI, FITC and DAPI/FITC, respectively. The images (**a**–**f**) are representative of 40 images observed in three independent experiments.

**Figure 4:**
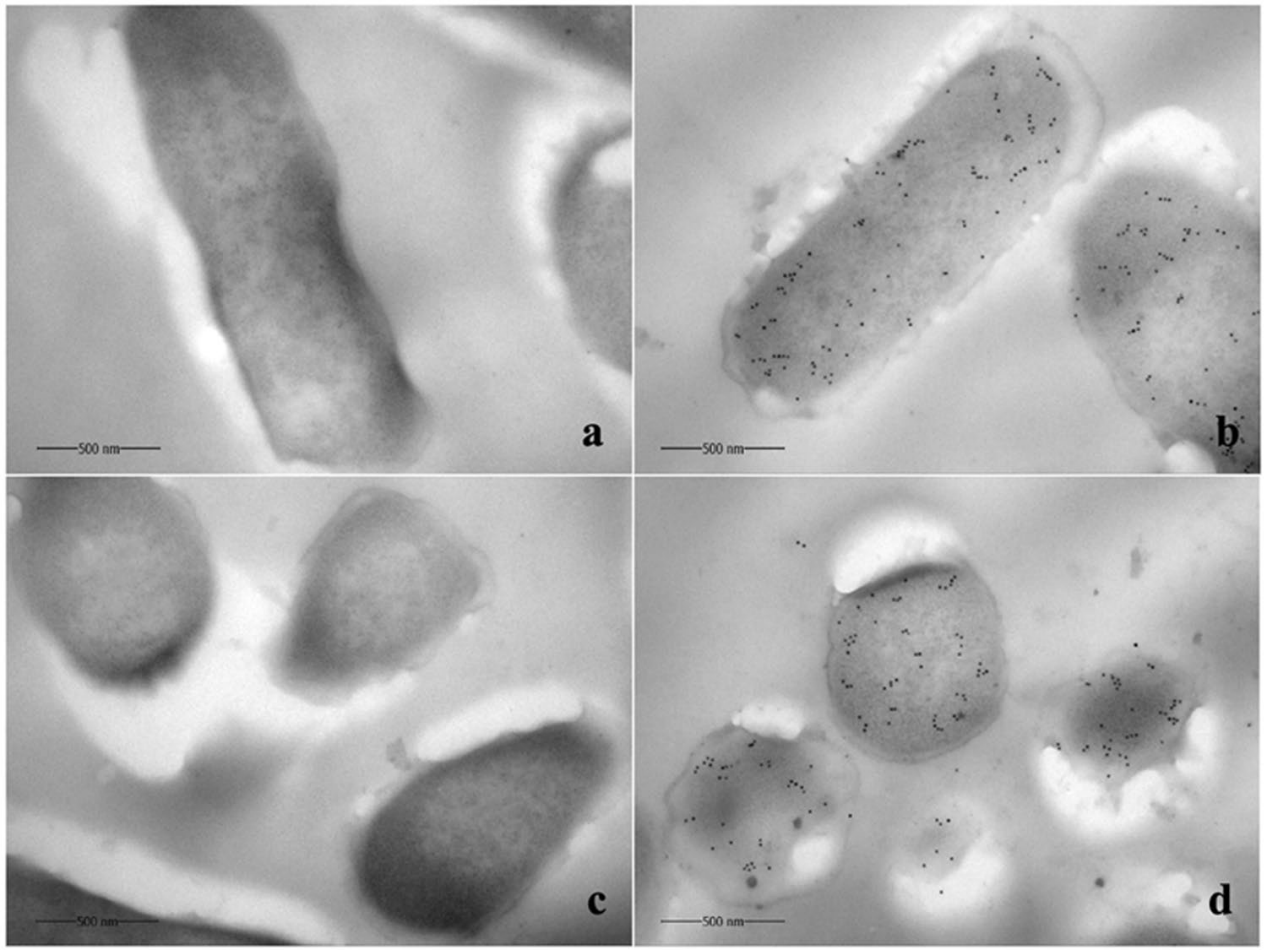
Analysis of *E*. *coli* after treatment with pBD2 for 4 h by immuno-gold transmission electron microscopy. (**a**,**c**) Are control cells from longitudinal sections and cross-sections, respectively; (**b**,**d**) Show *E*. *coli* from longitudinal sections and cross-sections, respectively, treated with 150 μg/ml pBD2 for 4 h. The images (**a**–**d**) are representative of 52 images observed in two independent experiments.

Immuno-fluorescence analysis was used to determine pBD2 localization in bacterial cells (Fig. 3). The results showed higher concentration of pBD2 on bacterial membranes at both ends of *E*. *coli*, and some of the pBD2 peptide was localized inside the cells, suggesting penetration of pBD2 into bacterial cells. Moreover, immuno-gold TEM analysis was used to confirm the precise localization of the peptide in bacterial cells (Fig. 4). *E*. *coli* cells analyzed after culturing with 150 μg/mL pBD2 for 4 h demonstrated collapsed cell membranes and cytoplasmic localization of pBD2, as evidence by the presence of gold particles mainly in the cytoplasm (Fig. 4b,d). Our results showed several other striking structural alterations in *E*. *coli* treated with pBD2 including indistinct inner membranes in many regions, loss of the double membrane structure and cell contents from the damaged membranes, particularly from the cross-section (Fig. 4c,d). The results from both approaches demonstrated that pBD2 was possibly located on the membrane and mainly in the cytoplasm of *E*. *coli*.

### Identification of DEGs by ACP-based RT-PCR

**Figure 5:**
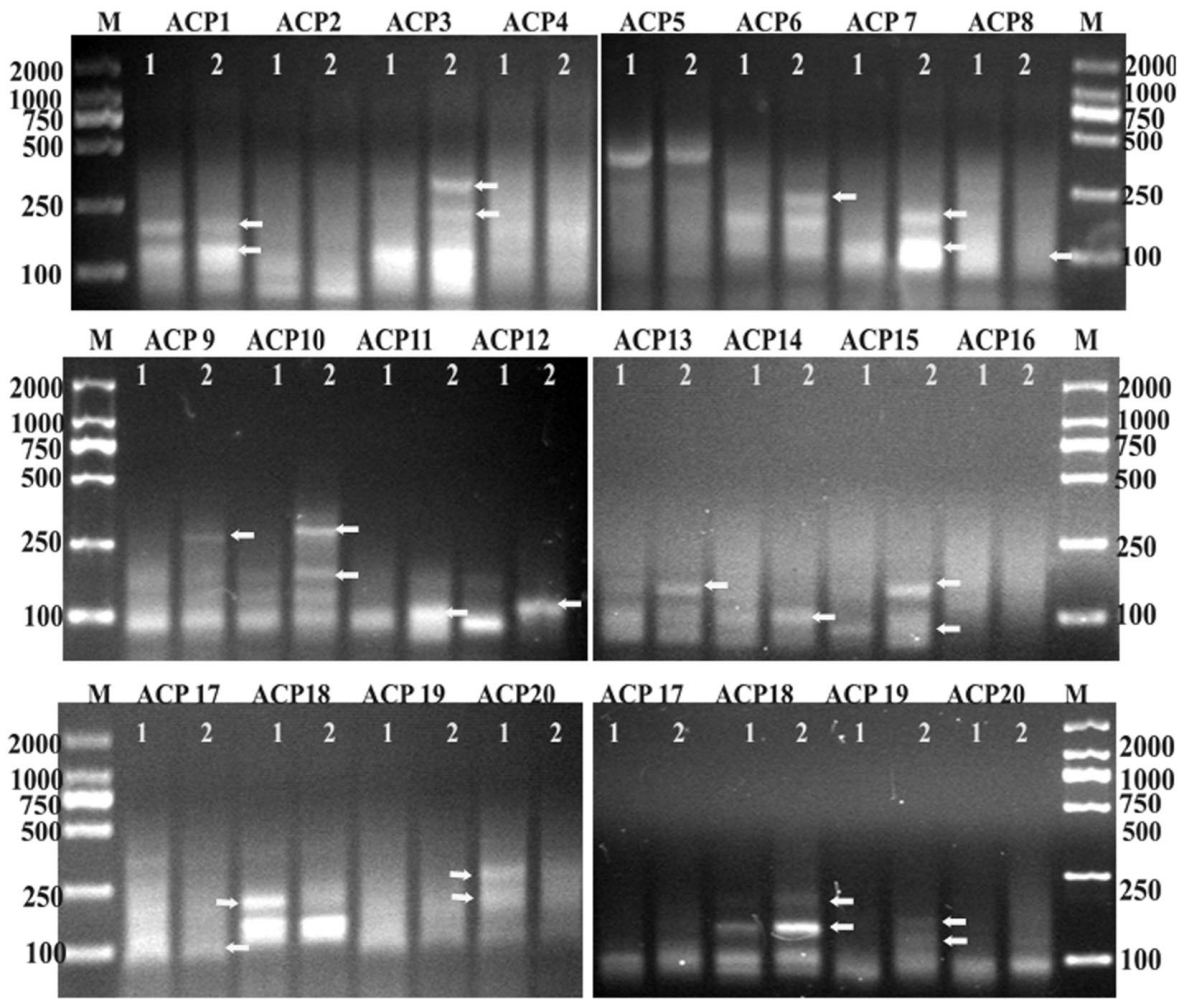
The identification of DEGs between control and pBD2-treated *E. coli* using the annealing control primer (ACP)-based PCR method by agarose gel electrophoresis. Agarose gel electrophoresis shows some part of the DEGs between the control (lane 1) and pBD2-treated *E*. *coli* (lane 2) using 20 arbitrary ACPs. The arrows indicate the DEGs. M indicates DNA marker DL2000. The full-length gels are presented in Supplementary Figures 2–7.

Spectrophotometric analysis of RNAs extracted from treated and untreated *E*. *coli* showed that the OD_260_/OD_280_ ratios were between 1.8 and 2.2 and that concentrations were above 1 μg/μL, suggesting the high purity and optimum quantity of RNA suitable for further downstream analysis. Using 20 arbitrary primers with ACP-based RT-PCR analysis, the results showed a variety of differential gene expression among control and pBD2 treatment groups. As the patterns of all the PCR gels were similar at different pBD2 concentrations and times for each arbitrary primer, except for a few DEGs that were different in different treatments, a subset of the results is shown in Fig. 5. The DEGs were cloned into a T-Vector and sequenced. From the 20 tested arbitrary primers, more than 160 sequences were obtained, and more than 90 sequences were successfully sequenced.

### Analysis of the DEGs

The sequences were analyzed by BLAST analysis using the NCBI and ENSEMBL databases. The results showed that 38 transcripts were differentially expressed in response to pBD2 treatment and that 27 of them corresponded to 28 functional genes (one sequence covers two genes); 7 fragments were between two genes; and 4 were unknown functional protein sequences (hypothetical proteins). The results are listed in Table 1. Twenty-eight known DEGs are classified, abbreviated and listed in Table 2. Among the 28 known functional DEGs, 6 genes were related to ion transport or protein trans-membrane transport, all of which are membrane proteins; 13 genes were related to DNA transcription and translation (including two fragments coding tRNA), all of which are in the cytoplasm; and 9 genes were related to metabolism, where among them, 1 gene is a membrane protein, 3 genes are in the cytoplasm, and 5 genes have been predicted to be in localized in the cytoplasm according to their functions. These results suggested that among the 28 known functional genes, 7 genes regulate the function of cell membranes, and 21 genes play an important role in the cytoplasm.

**Table 1:** The differentially expressed fragments by ACP-based RT-PCR technology.

Name^a^	ACP-based PCR	Size (bp)	Protein
37E1h 3-1	up	90	Phosphate ABC transporter substrate-binding protein/phosphate transporter subunit.
37E1h 4-2	down	11	Ferrichrome transport ATP-binding protein FhuC/iron-hydroxamate import ATP-binding protein.
37E4h 2	up	74	Chloramphenicol acetyltransferase.
37E4h 11	up	51	LPS core phosphoethanolamine transferase, integral component of membrane.
37E4h 20-1	down	33	Succinate-CoA synthetase, beta subunit.
37E4h 20-2	down	60	Soluble pyridine nucleotide transhydrogenase.
75E1h 3-1/3-2/150E 1h 3-1/150E4h 3-1/150E4h 5-1	down	67/43/98/47/107	10Sa stable RNA/transfer-messenger RNA/SsrA RNA
75E1h 9-1/75E4h 9	up	346/78	Preprotein translocase, SecY subunit/putative ATPase subunit of translocase
75E1h 9-2	down	29	Ethanolamine ammonia-lyase large subunit.
75E4h 15/150E4h 14-4	up	70/109	2-polyprenyl-6-methoxyphenol 4-hydroxylase.
75E4h 19	up	30	50 S ribosomal protein L14.
150E1h 5-3	up	126	Lysine tRNA synthetase.
150E1h 6-1/6-2	up	102/59	Regulatory RNA CsrB (carbon storage regulator B).
150E1h 6-3	up	27	DL-methionine transporter subunit.
150 E1h 10/(150E4h 1/10-1/10-2)	down/up	197/(70/65/58)	23S rRNA.
150E1h 15-1	up	23	Lactose operon repressor.
150E1h 15-2	up	592	Sequence cross two genes, the first is S-(hydroxymethyl)glutathione dehydrogenase/class III alcohol dehydrogenase, the second is S-formylglutathione hydrolase (42 bp), there are 93 noncoding bp between them.
150E1h 19-1	up	300	Transcriptional regulator Cbl/HTH-type transcriptional regulator cbl.
150E1h 19-2/150E4h 19-1	down	57/58	Phosphohistidine phosphatase, in cytoplasm.
150E4h 2-2/3-4/14-3	up	41/52/63	Trehalose-6-phosphate phosphatase.
150E4h 7-1	down	22	Coding tRNA between 16 S rRNA and 23 S rRNA.
150E4h 7/9-4/75E 4h 7-2	up	23/81/30	Coding tRNA between 16 S rRNA and 23 S rRNA (different from tRNA above).
150E4h 9-2	up	50	Lipid transporter ATP-binding/permease, integral component of membrane, ATP-binding, the main processes involved in transmembrane transport.
150E4h 9	up	144	Potassium transporting ATPase subunit B, integral component of membrane.
150E1h 14-1	up	49	DNA-directed RNA polymerase, beta subunit.
150E4h 15	up	374	Histidyl-tRNA synthetase.
150E4h 20-1	down	92	16 S rRNA (cytosine(1402)-N(4))-methyltransferase.
75E1h 1-2	down	68	Hypothetical protein 1 (645 bp 214 aa), function is unknown.
75E1h 9	up	120	Hypothetical protein 2 (408 bp 135 aa), linked to flavinator of succinate dehydrogenase family proteins.
75E4h 2-1	down	42	Hypothetical protein 3(417 bp,198 aa), associated with bacterial flagellum assembly.
150E1h 20	down	41/135	Hypothetical protein 4 (207 bp,68 aa), function is unknown.
37E1h 3-2	up	45	Sequence 1 between two genes, one is a phospholipase D family protein, the other is a hypothetical protein (HP6, 981 bp, 326 aa) .
37E4h 2-1	up	74	Sequence 2 between two genes, one is methylenetetrahydrofolate reductase, the other is catalase/peroxidase HPI.
150E1h 3-2	up	67	Sequence 3 between two genes, one is protein CsiD, the other is SsrA-binding protein.
150E1h 6-2/7-2	up	103/194	Sequence 4 between two genes, one is hypothetical protein (HP 5, 330 bp,109 aa), the other is protein syd.
150E1h 17	down	272	Sequence 5 between two genes, one is glycerate kinase 2, the other is bacterial regulatory helix-turn-helix, lysR family protein.
150E4h 3-1	up	259	Sequence 6 between two genes, one is a TonB-dependent siderophore receptor family protein, the other is serine protease sat auto-transporter.
150E4h 9-5	up	37	Sequence 7 between two genes, one is citrate lyase acyl carrier protein (18 bp within it), the other is citrate (pro-3S)-lyase ligase.

Note: The first number of the name represents the co-cultured pBD2 concentration (37.5, 75,or 150 μg/mL); E is the abbreviation for *E*. *coli*, 1 h or 4 h indicted for co-culture of 1 h or 4 h. The last number indicates the random primers in the GeneFishing kit and the amplified band.

**Table 2:** The functions, locations and abbreviations of DEGs.

Name	Main functions	Locations	Abbreviations
**Transporter**			
potassium transporting ATPase subunit B	potassium transport, this subunit is responsible for energy coupling to the transport system	integral component of membrane	kdpB
ferrichrome transporter ATP-binding protein FhuC	involved in iron(III) hydroxamate import, responsible for energy coupling to the transport system	peripheral proteins of the cytoplasmic membrane	FhuC
preprotein translocase subunit SecY	the central subunit of the protein translocation channel SecYEG	integral component of membrane	SecY
DL-methionine transporter subunit	involved in methionine import	integral component of membrane	metI
Lipid transporter ATP-binding/permease	involved in lipid A export and for biogenesis of the outer membrane	integral component of membrane	msbA
Phosphate ABC transporter substrate-binding protein	involved in phosphate import	integral component of membrane	pstS
**Metabolism**			
succinyl-CoA synthetase, beta subunit	carbon metabolism, energy metabolism, citric acid cycle(TCA cycle)	in cytoplasm	sucC
trehalose-6-phosphate phosphatase	glucose metabolism, osmotic adaptation	unknown, perhaps in cytoplasm	otsB
alcohol dehydrogenase	energy metabolism, carbon fermentation	in cytoplasm	frmA
S-formylglutathione hydrolase	hydrolyzes S-formylglutathione to glutathione and formate	unknown, perhaps in cytoplasm	frmB/YeiG
LPS core phosphoethanolamine transferase	catalyzes the addition of a diphosphoethanolamine moiety to the outer membrane lipopolysaccharide core	integral component of membrane	eptC
soluble pyridine nucleotide transhydrogenase	pyridine nucleotide-disulfide oxidoreductase family protein, has an impact on the redox balance of cells	in cytoplasm	udhA/sthA
ethanolamine ammonia-lyase, large subunit	intracellular amino acid metabolism	unknown, perhaps in cytoplasm	eutB
chloramphenicol acetyltransferase	transport of small molecules	unknown, perhaps in cytoplasm	cat
2-octaprenyl-6-methoxyphenyl hydroxylase	FAD/NAD(P)-binding protein	unknown, perhaps in cytoplasm	ubiH
**DNA transcription and translation**			
23S rRNA	ribosomal component, protein synthesis	in cytoplasm	23S rRNA
50S ribosomal protein L14	ribosomal proteins, protein synthesis	in the ribosomal	rplN
lysine tRNA synthetase	tRNA synthesis, protein synthesis	in cytoplasm	lysS
histidyl-tRNA synthetase	tRNA synthesis, protein synthesis	in cytoplasm	hisS
10saRNA	transcriptional regulator	in cytoplasm	10SaRNA
lactose operon repressor	transcriptional regulator, repressor of the lactose operon	in cytoplasm	lacI
transcriptional regulator Cbl	HTH-type transcriptional regulator, transcriptional regulator of cysteine biosynthesis	in cytoplasm	cb1
regulatory RNA CsrB	control bacterial gene expression post-transcriptionally	in cytoplasm	CsrB
phosphohistidine phosphatase	catalytic diphosphoric acid reaction	in cytoplasm	sixA
DNA-directed RNA polymerase, beta subunit	transcription	in cytoplasm	rpoB
16S rRNA (cytosine(1402)-N(4)) -methyltransferase	catalyzes the N^4^-methylation of cytosine in 16sRNA	in cytoplasm	rsmH
coding tRNA between 16sRNA and 23sRNA	coding tRNA	in cytoplasm	tRNA
**Protein besides sequences**			
phospholipase D family protein	related to the metabolism of phospholipids	unknown, perhaps in cytoplasm	phD
methylenetetrahydrofolate reductase	involved in the tetrahydrofolate pathway interconversion, which is part of one-carbon metabolism	in cytoplasm	metF
catalase/peroxidase HPI	bifunctional enzyme with both catalase and broad-spectrum peroxidase activity	unknown, perhaps in cytoplasm	katG
protein CsiD	combined with Fe^2+^	unknown, perhaps in cytoplasm	csiD
SsrA-binding protein	binds to tmRNA, and required for rescue of stalled ribosomes	in cytoplasm	smpB
protein syd	interacts with the SecY protein *in vivo*	in membrane	syd
glycerate kinase 2	glucose metabolism	unknown, perhaps in cytoplasm	gk2
bacterial regulatory helix-turn-helix, lysR family protein	combine with DNA, regulated transcription	in cytoplasm	lysR
TonB-dependent siderophore receptor family protein	transport, related to siderophore transport	in outer membrane	TonB
serine protease sat autotransporter	has serine-type endopeptidase activity	in outer membrane	sat
citrate lyase acyl carrier protein	covalent carrier of the coenzyme of citrate lyase	in cytoplasm	citD
citrate (pro-3S)-lyase ligase	ligase	unknown, perhaps in cytoplasm	citC

Among the 6 ion or protein transport genes, kdpB, FhuC and pstS are related to ion transport, secY and metI are related to protein translocation channels and methionine import, and msbA is involved in lipid A export and for biogenesis of the outer membrane. Among the 9 metabolic genes, sucC, otsB and frmA are related to carbon metabolism, and frmB and eutB are related to amino acid metabolism. eptC is related to LPS core biosynthesis. udhA and ubiH are related to redox balance; udhA has an impact on the redox balance of cells, which could convert NADPH to NADH, through which it is involved in the respiratory chain for energy generation. ubiH has oxidoreductase activity, acting on paired donors, with incorporation or reduction of molecular oxygen. Thirteen genes are related to DNA transcription and translation, and they are all located in the cytoplasm. 10SaRNA, CrsB, cb1, lac1, sixA and rsmH are all related to regulation of DNA transcription. 10SaRNA (also known as SsrA RNA or tmRNA) has properties of tRNA and mRNA; it binds to SsrA-binding protein (the gene is adjacent to sequence 5) and rescues stalled ribosomes, known as trans-translation. CrsB is a non-coding RNA molecule that antagonizes the effects of CsrA *in vivo*. CsrA modulates glycogen synthesis and catabolism, among other process, cb1 regulates two operons consisting of ABC transporters that are part of the cys regulon and is an ssuEADCB and tauABCD operon transcriptional activator. 23sRNA, rplN, lysS, hisS and rpoB, and tRNA are involved in DNA translation or protein synthesis. There are 14 genes adjacent to the 7 differently expressed fragments; among them, there are 2 hypothetical proteins and 12 known functional genes (also listed in Table 2). They are also related to transport (TonB, sat and syd, in the membrane), DNA transcription and translation (lysR and smpB, in the cytoplasm), and metabolism (phD, metF, katG, csiD, gk2, citD and citC). Among the 4 unknown genes, hypothetical protein 3 (HP3, 417 bp, 198 aa) is known to be associated with bacterial flagellum assembly, and HP2 (408 bp, 135 aa) is linked to the flavinator of succinate dehydrogenase family proteins.

### Quantitative real-time PCR confirmation for selected genes

**Figure 6:**
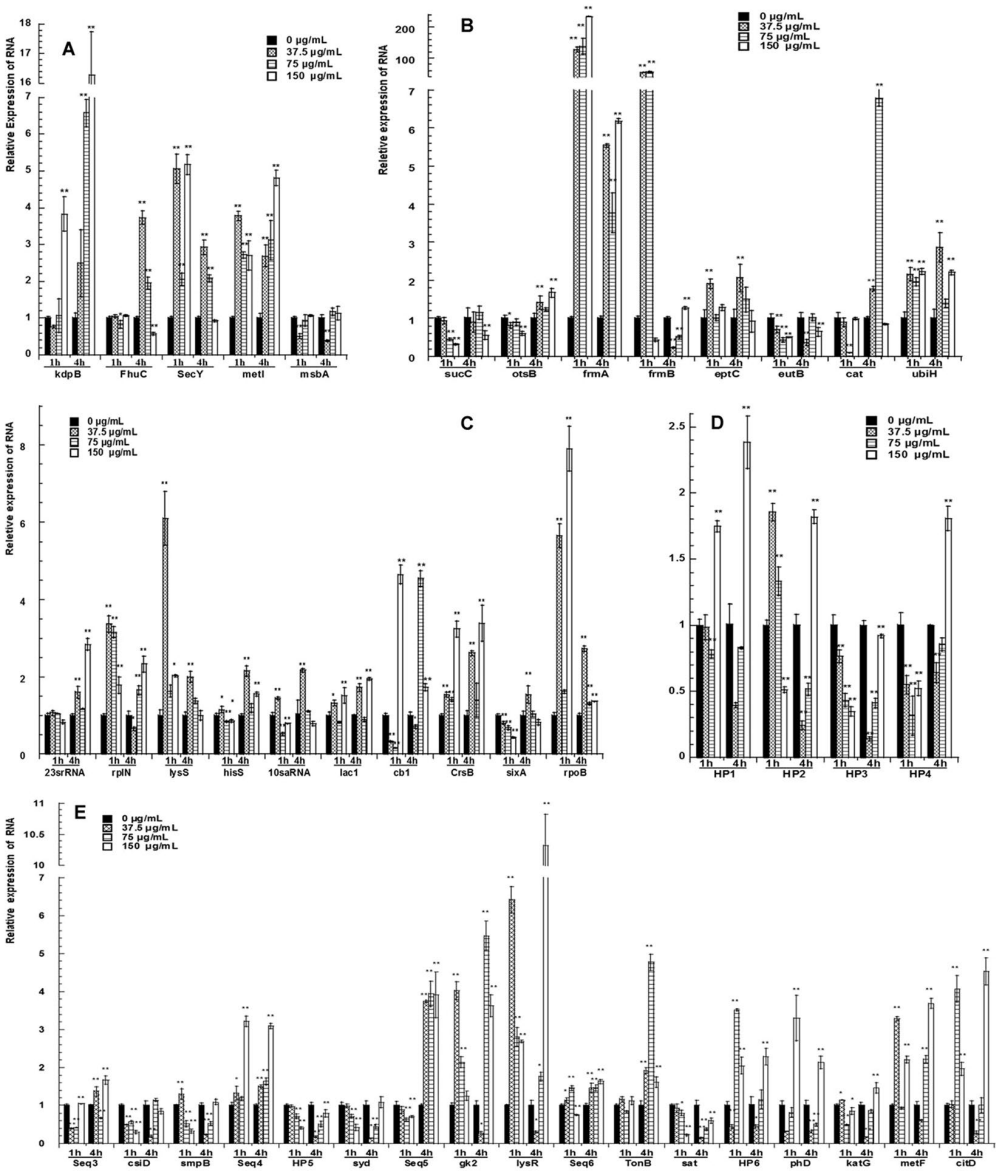
Quantitative real-time PCR confirmation for selected genes. qRT-PCR was performed with SYBR Green Dye (TaKaRa, Dalian, China) using a Bio-Rad CFX96 real-time PCR platform. All the samples were analyzed in triplicate and fold-changes of gene expression were calculated by 2^−△△CT^ methods with GAPDH as a reference gene. The * and ** at the top of column indicate the significant differences from the control at *P* < 0.05 and *P* < 0.01, respectively.

qRT-PCR was used to confirm the DEGs of *E*. *coli* in response to different concentrations of pBD2 treatments. The relative expression level of each gene was normalized by GAPDH gene expression. The results of qRT-PCR are consistent with the results of ACP-based RT-PCR and are shown in Fig. 6. The expression analysis of transport genes is shown in Fig. 6A. The genes secY and metI were upregulated 2- to 16.2-fold, respectively, in all the treatment groups compared to that of the control (*P* < 0.01) at 1 h and 4 h post treatment with the exception of secY gene expression at 4 h, which had no change with the maximum dose of pBD2 treatment. kdpB expression was higher at 1 h (*P* < 0.01) in response to only the highest concentration (150 μg/mL) of the pBD2 treatment but was consistently upregulated at 4 h in all the treatment groups. Expression of msbA was reduced in *E*. *coli* cells treated with 37.5 μg/mL of pBD2 compared to that of the untreated control (*P* < 0.01). The expression of Fhu differed at 1 h and 4 h. As shown in Fig. 6B, the levels of metabolism-related genes frmA and ubiH were increased, while sucC and eutB transcripts were basically decreased in response to pBD2 treatment. otsB was downregulated at 1 h and upregulated at 4 h (*P* < 0.05 and *P* < 0.01, respectively). eptC was upregulated in response to the 37.5 μg/mL pBD2 treatment (*P* < 0.01), and frmB and Cat genes were altered at different pBD2 concentrations and at different times. Expression analysis of genes involved in DNA transcription and translation are shown in Fig. 6C. Expression of 23srRNA, rplN, lysS, lac1, CrsB and rpoB was basically 1- to 8-fold upregulated (*P* < 0.05 or 0.01) in pBD2 treated-cells compared to that of the control. cb1 expression was lower at lower pBD2 concentrations (*P* < 0.05) and higher at high concentrations (*P* < 0.01). sixA was downregulated at 1 h and upregulated at low pBD2 concentration at 4 h (*P* < 0.01). In total, the proteins for DNA transcription and translation were influenced. Four hypothetical proteins are shown in Fig. 6D. HP3 was downregulated (*P* < 0.01). Interestingly, the expression level of HP3 was decreased gradually at 1 h and increased at 4 h with increased pBD2 concentrations. HP1 was upregulated at 150 μg/mL pBD2 treatment (*P* < 0.01). HP2 and HP4 varied at different times and concentrations.

The longer sequences between two genes were selected and the expression levels were detected, including the two genes shown in Fig. 6E. Sequences 4 and 6 were basically upregulated (except for seq6 at 150 μg/mL for 1 h); sequence 5 was downregulated at 1 h and upregulated at 4 h (*P* < 0.01). Sequence 3 varied at different times and concentrations and was significantly different from the control (*P* < 0.01). The changes in two genes (HP5 and syd) near sequence 4 were downregulated (*P* < 0.01); moreover, the trends of their changes were consistent. The trends of transcript change in gk2 and lysR other than seq5 were also consistent, as was the case for HP6 and phD other than seq1, and katG and metF other than seq2. In total, the genes nearby besides differentially expressed fragments were changed themselves.

In summary, the results of qRT-PCR confirmed the results of ACP-based RT-PCR.

## Discussion

pBD2 is one of the defensins secreted by pigs and shows high antibacterial activity against bacteria, making it a good candidate for antibiotics^15,16^. Veldhuizen (2008) reported that synthetic pBD2 showed high antibacterial activity against pathogenic intestinal bacteria and could inhibit *E*. *coli*, *S*. *typhimurium*, and *P*. *aeruginosa* and so on^16^. Our previous reports showed that pBD2 had high antibacterial activity against gram-negative *E*. *coli* and gram-positive *S*. *aureus*, including multi-resistant *E*. *coli* isolated from fowl^15^. The bacterial concentration for tests was usually approximately 10^4^–10^6^ cfu/mL; in this test, the concentration was raised to 1 × 10^9^ cfu/mL because a large number of bacteria were required in the later experiments. The results showed that expressed pBD2 could inhibit the growth of 10^9^ cfu/mL bacteria. In addition, antibacterial activity was increased with pBD2 concentration and decreased with exposure time (*P* < 0.01), which was consistent withprevious results for 10^5^–10^6^ cfu/mL bacteria in our lab^15^.

After co-culture for 1 h and 4 h with different pBD2 concentrations, morphological damage occurring in response to different doses of pBD2 was similar. *E*. *coli* was seriously damaged and showed pronounced changes including leakage of contents and debris from cell death (Fig. 2), and the damage was more serious with increased pBD2 concentrations and exposure times. Some cells death appeared to be because of the cell contents leaking (Fig. 2C), some cells appeared to be burst with deep craters (Fig. 2B,F) and some cells deposited very small pieces of cell debris, appearing that the cells were dissolved (Fig. 2D,G). These results may imply that there is more than one mechanism causing cell death. Moreover, some white particles or blebs appeared on the cell surface (Fig. 2B,D), and it was difficult to distinguish whether they were either debris from burst cells or blebs because of cell damage from pBD2. In addition, pBD2 may have killed the *E*. *coli* one by one because some cells appeared normal, and other cells were dead in the same field of view. Some morphological changes of *E*.*coli* in this experiment are consistent with those of *E*. *coli* effected by human epididymis 2 (HE2) protein isoforms, HE2α/HE2β1/HE2β2^32^. However, our results differed from those for antimicrobial peptides gramicidin S (GS) and PGLa acting on *E*. *coli* in hypotonic medium on silicon platelets^33^. Our results showed morphological changes were more complex, implying that the actions of pBD2 might be complex, which requires further research.

The location of pBD2 in *E*. *coli* was detected by IFM. The results indicated that pBD2 was distributed on the membrane, specially focused at both ends of *E*. *coli*, and that some was found inside of the bacteria. The locations of pBD2 in bacteria cells were further confirmed by immune-gold TEM, which demonstrated that pBD2 was a cell-penetrating peptide. Antimicrobial peptide magainin 2, chicken cathelicidin-2, and SpHyastatin among others have been reported to pass through the cell membrane and are located in the cytoplasm of bacteria^34–38^. Wang *et al*. have reported that a marine arenicin-3 derivative, N4, permeabilized the outer membrane of *E*. *coli* within 1 min then disrupted the plasma membrane and entered the cytoplasm after 0.5 h^39^. Wei *et al*. have reported that the antimicrobial peptide buforin II (BF2), and HipC (a cell-penetrating peptide) entered into the majority of spheroplasts, while BF2 with a P11A mutation and magainin localized with the membrane^40^. Nan *et al*. reported that the antimicrobial peptide indolicidin (IN) and its analogs IN-1 and IN-2 did not enter the *E*. *coli cell* membrane, whereas IN-3 and IN-4 penetrated the membrane^25^. These results indicate that AMPs have different mechanisms, even when they have similar structures. Our results indicated that pBD2 was a cell-penetrating peptide and could enter the *E*. *coli* cells, perhaps targeting intracellular molecules and playing important roles in the cytoplasm. In addition, *E*. *coli* undergoes fission, starting from one end of the bacteria, which may be the most easily invaded location by defensins, and this may be the reason why pBD2 mainly focused on the ends of the *E*. *coli*. These results demonstrated that pBD2 could disrupt the membrane and enter the cytoplasmic membrane.

It is well known that the first step for antimicrobial peptides to kill bacteria is interacting with the negatively charged lipids on the membrane surface. We had predicted the structure of mature pBD2, composed of 37 amino acids, based on the structure of hBD1. The peptide has a typical beta defensin shape with one alpha helix (His2-Lys8) and three beta sheets (Thr11-Asn13, Phe20-Tyr28 and Lys31-Arg37). The structure of recombinant pBD2 with a His-tag was predicted to have another alpha helix in its N-terminal^15^. The three disulfide bonds make the peptide flexible and impart a stabilizing force. These stable structures are more likely to disrupt surface tension and create membrane defects that ultimately break down the bilayer integrity of bacteria^30^. pBD2 may interact with the outer membrane and then may either aggregate on the membrane or, once a critical concentration is reached, lead to the disruption of the membrane, followed by pBD2 interacting with the inner membrane and entering into the cells.

In this study, RNAs of *E*. *coli* were extracted according to the reported method with some modifications^41^, and ACP-based RT-PCR without adding poly(A), DEGs were found. The results proved that the prokaryotic RNA contained polyadenylic acid. To obtain polyadenylated RNA, the RNAs were not isolated by conventional procedures involving deproteinization by phenol extraction, which appeared to cause specific losses of poly(A)-RNA^41^. ACP-based RT-PCR technology is an easy differential display technology (detected by agarose gel electrophoresis). The core of the technology is an annealing control primer, which binds highly specifically to the target template and can provide greater annealing specificity during PCR^42^. The system is used to identify DEGs without generating false positives. Therefore, this technique has been widely used to identify DEGs under different treatment conditions and for the development of organisms^43–45^. By ACP-based RT-PCR, the DEGs were detected and were very samilar in response to different pBD2 concentration treatments and exposure times with each annealing control primer, which indicated ACP-based RT-PCR was credible and repeatable. By this technique, 38 DEGs were obtained, and 28 known genes from these were analyzed. Most changed genes were related to DNA transcription and translation, and mainly were in the cytoplasm. These results suggest that the pBD2 influenced genes mainly in the cytoplasm and with DNA transcription and translation functions. A great deal of researches has indicated that AMPs act with intracellular molecules, such as DNA or ribosomes, which can subsequently result in influence on DNA or RNA synthesis, such as PR-39, indolicidin and its analogs, buforin 2 and its anologues BF2-A/C and so on^21,23–25,46^. Most of the DEGs of *E*. *coli* were in the cytoplasm and related to DNA transcription and translation, and the results implied that pBD2 kills *E*. *coli* by influencing DNA or RNA through the cell membrane, which is consistent with the location of pBD2. Among the 28 known genes besides the genes in the cytoplasm, 7 known genes on the membrane were influenced, and they are mainly integral components of the membrane and related to transport of potassium ions, iron ions, phosphate ions, and so on. In addition, the two genes next to seq6 were involved in transport. These results suggest that transport proteins in the membrane also changed, and there have been some reports that AMPs killed bacteria based on the ion mechanism^47–49^. The genes for metabolism were changed, along with those involved in carbon (energy) metabolism, and amino acid and redox balance, which are all involved in the actions of AMPs killing bacteria^29^.

The results of qRT-PCR confirmed the results of ACP-based RT-PCR. The expression of membrane transporters was basically increased in *E*. *coli*. Expression of kdpB changed, and suggesting that potassium ion balance was disrupted. Yang *et al*. have reported that human beta defensin 2 (hBD2) is able to inhibit potassium channels^47^, and perhaps pBD2 has this activity, leading to higher kdpB levels. Ferric siderophore receptors provide an advantage for the cells to scavenge iron but can also be exploited by antibacterial compounds^48^. In *E*. *coli*, antibacterial peptide MccJ25 can bind outer membrane ferric siderophore receptor FhuA and enters the target bacteria and kills them through a TonB-dependent process^48,49^. FhuC, one of the ferric siderophore receptors, and the TonB-dependent siderophore receptor family protein (TonB) next to sequence 6 were upregulated at 4 h, which implied that pBD2 killed bacteria by influencing these processes. Protein CsiD combined with Fe^2+^, which had lower expression, implied that perhaps soluble ferrous iron (Fe^2+^) was in short supply, was oxidized to insoluble ferric iron (Fe^3+^) and was associated with the high expression of Fhu^50^. Meanwhile, SecY and metI were also upregulated, which suggests that translocation of the proteins and amino acids changed. Expression of msbA, involved in the transport of lipid A across the inner membrane and for biogenesis of the outer membrane, was lower than that of the control, suggesting that the biogenesis of membranes was influenced, potentially inhibiting the growth of *E*. *coli*. The genes for metabolism changed in different ways: expression of frmA, which influences energy metabolism, changed more than 100-fold compared to that of the control at 1 h pBD2 treatment, which implied that the energy station may be deeply affected, including the changes in sucC and otsB. Antimicrobial peptide LL-37 was reported to killing the bacteria mainly by target energy metabolism^29^. eptC is related to LPS core biosynthesis and had high expression at low pBD2 concentration and lower expression at high concentration, implying that bacteria may survive at low pBD2 concentrations. ubiH has oxidoreductase activity, and its high expression implied that the redox balance was disrupted.

The changed genes for DNA transcription/translation were mainly affected in response to pBD2. rplN, lysS and rpoB were upregulated, which enhance DNA transcription. Moreover, upregulated CrsB also leads to enhanced DNA transcription. CrsB antagonizes the effects of CsrA *in vivo*, and CsrA can facilitate *glgC* (glycogen biosynthesis) mRNA decay *in vivo* and inhibit *in vitro glg* gene expression posttranscriptionally^51^. The functions of 10sa RNA and smpB (SsrA-binding protein, the gene next to sequence 3, Fig. 6F) were to rescue the stalled ribosomes^52,53^. Their high expression at low pBD2 concentrations at 1 h implied that bacteria may be able to rescue the stalled ribosomes caused by pBD2 exposure, while lower expression at higher pBD2 concentration implied that the ribosomes might be inactivated. Roy *et al*. reported that proline-rich peptides, such as Bac7_1–35_, oncocins, and two oncocin derivatives, blocked the peptide exit tunnel of the ribosome and interfered with the initiation step of translation, which led to inactivated protein synthesis^54,55^. Florin *et al*. reported that Api137, a derivative of the insect-produced antimicrobial peptide apidaecin, bound the *Escherichia coli* ribosome and resulted in a global shutdown of translation termination^56^. sixA was apparently downregulated at 1 h, which enhanced DNA transcription, while its lower expression (at 37.5 μg/mL at 4 h) inhibited related gene expression. cb1 regulates two operons consisting of ABC transporters, and its high expression at higher concentration enhanced gene expression, while its lower expression at lower pBD2 concentration inhibited expression. Our results appear contradictory; it is difficult to conclude that pBD2 enhances or reduces gene expression, perhaps indicating that genes related to DNA transcription and translation had dynamic changes in response to pBD2 concentrations and exposure times, which requires further study.

The noncoding sequences including the nearby genes were also changed. In addition, those genes are related to transport, DNA transcription and translation, and metabolism. TonB, sat and syd were related to transport. TonB was related to siderophore transport, similar to FhuC, and their expression levels were basically consistent. Protein Syd interacts with the SecY protein *in vivo* as a regulatory factor that negatively controls the translocase function of SecY, and their expression levels was inversely related (Fig. 6). The lysR family protein also regulated transcription and had high expression, which implied that DNA transcription was influenced. phD, metF, katG, csiD, gk2, citD and citC are related to metabolism; phD is related to the metabolism of phospholipids, and its expression level was lower at lower pBD2 concentrations and higher at higher concentrations, which indicates that pBD2 may influence membranes at different concentrations. citD and citC are related to citrate lyase; citD had high expression and is related to glucose metabolism, providing energy, same to gk2. Although there is not a clear connection between noncoding sequences and their nearby genes, the nearby genes were clearly changed, and the noncoding sequences may have regulated the expression of their nearby genes. The detected seq5 and seq6 are longer than 200 bp and may be long non-coding RNA, and their function requires further study. In short, from differential gene analysis, pBD2 affects the transport of cell membranes, DNA transcription/translation and metabolic activities. Specifically, the main effect is on DNA transcription and translation.

From the analysis above, pBD2 has multiple actions. It disrupted cell membranes, influenced the ion and energy balance, and affected DNA transcription and translation. Overall, the main action of pBD2 is the influence on DNA transcription and translation by targeting the intracellular molecules after membranes are damaged.

## Conclusion

After incubation with pBD2, the contents of *E*. *coli* leaked. pBD2 was located in the cell membrane and cytoplasm based on IFM and predominantly in the cytoplasm according to immuno-gold TEM. DEGs of *E*. *coli* were detected by ACP-based RT-PCR, and they were related to transport and transmembrane transport proteins in the membrane and DNA transcription and translation and metabolic proteins in the cytoplasm. The known functional DEGs were mainly related to DNA transcription and translation. The results of qRT-PCR confirmed those of ACP-based RT-PCR; the transmembrane transport proteins were mainly upregulated; and the genes related to DNA transcription and translation were influenced. The main mechanism for pBD2 killing *E*. *coli* is the influence on DNA transcription and translation by entry into the cytoplasm after membranes are damaged, although other proteins are also affected. The results explained the mechanism of pBD2 killing *E*. *coli*, which will be the basis for further study and for the application of this AMP in pig disease.

## Materials and Methods

### Bacterial strains and growth conditions

*E*. *coli* ATCC 25922 used in the present study was purchased from the Beijing Ordinary Microbiology Strain Store Center, Beijing, China. The *E*. *coli* were cultured in Luria broth (LB) medium (1% peptone, 0.3% beef extract, and 0.5% NaCl) overnight and then inoculated into fresh LB medium for 2–4 h with constant shaking at 220 rpm.

### Preparation of pBD2

The engineered strain *E*. *coli* BL-pET-*pBD2* was cultured and induced according to the procedures described previously^15^. In brief, *E*. *coli* BL-pET-*pBD2* was induced by 1 mM IPTG for 4 h and was lysed by sonication for 30 min. The recombinant pBD2 was purified by a Ni-NTA agarose column (His-tag affinity column) and dialyzed against 10 mM phosphate buffer or water and concentrated with polyethylene glycol 8000. The concentration of protein was determined by the BCA method.

### Kill kinetics

The kill kinetics were performed by the turbidimetric method as reported previously^15,57^. Optical density of the bacterial cells was measured at 600 nm, and the absorption value of OD_600_ was adjusted to approximately 1 (the final density was approximately 10^9^ cfu/mL). *E*. *coli* were incubated with pBD2 (final concentrations were 0, 37.5, 75, and 150 μg/ml) in polypropylene 96-well microtiter plates for different time durations (0 h, 1 h, 4 h, and 8 h). The optical density was measured at 630 nm (reference 405 nm) by a microplate reader (Stat Fax 2100, Awareness Technology Inc. USA). The OD value at 0 h was adjusted to 0, the increased OD values were calculated, and all assays were carried out in triplicate. All values were expressed as the mean ± standard deviation (SD).

### Bacterial treatments

*E*. *coli* at 10^9^ cfu/mL was suspended in different concentrations of purified recombinant pBD2 (0, 37.5, 75, and 150 μg/ml) at 37 °C for different lengths of time (1 h and 4 h), and bacteria were harvested after centrifugation at 3000 rpm for 5 min. After washing three times with 10 mM PBS, bacterial cells were used for RNA extraction, SEM, IFM and immuno-gold TEM.

### Scanning electron microscopy

SEM analysis was performed as described previously^33^. Briefly, *E*. *coli* cells were fixed with 2.5% glutaraldehyde in 0.01 M PBS buffer (pH 7.2) overnight at 4 °C. After fixation, cells were washed for 3 × 10 min in 10 mM PBS buffer and washed with distilled water twice. The samples were then dehydrated with gradient concentrations of ethanol and placed in ethanol and tert-butyl alcohol solution (1:1). The treated samples were placed in tin foil, dried in the air and gold-coated by an ion spray instrument (MSP-2S, IXRF, USA) to be analyzed by S-3400 scanning electron microscopy (Japan).

### Immuno-fluorescence analysis

Immuno-fluorescence analysis was performed according to methods published with some modifications^38^. *E*. *coli* cells were spread on anti-off slides (Wuhan Boster Biological Engineering Co., Ltd, China) and air-dried. After fixing with 4% paraformaldehyde for 2–4 h at 4 °C, the samples were treated with 0.5% Triton-100 in 10 mM PBS buffer for 15 min. Samples were then washed with 10 mM PBS buffer for 3 × 5 min and were incubated with 10% normal goat serum in PBS for 30 min at room temperate in a wet box. After drying with absorbent paper, bacterial samples were incubated with 50 µl polyclonal pBD2 (prepared by us from rabbit^58^, diluted to 1:500) at 4 °C overnight. Afterwards, cells were incubated with fluorescently (FITC) labeled secondary antibodies for 1 h at 37 °C in the box, washed with PBS for 3 × 5 min and then stained by DAPI solution for 5 min in the dark. After staining, samples were washed 3 times with PBS and blocking agent was added containing anti-fluorescence quencher and sealed with cover slips. Controls for immune-fluorescence were performed by incubating samples without primary antibodies. Cells were observed by microscopy (DM6000B, Leica, Germany).

### Immuno-gold transmission electron microscopy

Immuno-gold TEM was used to further determine the localization of the peptide as reported previously^36^. The cells were embedded in 2% low-melting point agarose, trimmed into small pieces (1 mm^3^), and fixed in fixative buffer for 2 h on ice. The samples were first dehydrated in 30% methanol for 15 min at 0 °C and then dehydrated with gradient concentrations of methanol at −20 °C. After infiltration in the mixtures of 100% methanol and Lowicryl K4M (the ratios were 1:1, 1:2, and 1:3) for 60 min and in pure Lowicryl K4M at −20 °C overnight. Samples were then embedded in gelatin capsules, filled with resin and capped tightly, polymerized under UV light (360 nm) and placed 20 to 30 cm away from the UV source for 48 h at −35 °C, after which they are placed for 12 h at room temperature.

The materials were cut into 0.1-μm thick sections by a CM1900 Frozen Slicer (Leica, Germany). Sections were mounted on 100 mesh copper Formvar-carbon coated grids and incubated with 10 mM PBS (pH 7.4) containing 0.05% Triton X-100, 0.05% Tween and 1% bovine serum albumin for 5 min at 20 °C. Further, sections were incubated with pBD2 antibody (1:200) for 2 h at RT and exposed to protein-A gold (1:50) for 1 h at RT, and specimens were then stained with 3% uranium acetate for 4 min and lead citrate for 1 min. For all electron microscopy, a JEM-1400 electron microscope was used. Cells incubated with PBS without primary antibodies were used as a negative control.

### RNA extraction of *E*. *coli*

The total RNA of *E*. *coli* was extracted according to the procedure reported previously with some modifications^41^. Briefly, 1 mL of cells at 10^9^ cfu/mL were washed with water and digested with lysozyme for 40 min in 15 mM Tris-HCl (pH 8.0), then protoplasts were collected and were lysed by 0.5% SDS in 80 mM Tris-HCl buffer (pH 7.5). Then, the proteins were removed by saturated sodium chloride solution. The RNA was precipitated and washed with 70% ethanol. After drying at room temperature, RNA pellets were then dissolved in RNase free water. The extracted total RNA was digested with DNase for 30 min and subjected to an ethanol-based purification before use in the downstream applications. The concentration and purity of RNA was determined by UV absorption at OD260 and OD280 by spectrophotometer (2800 UV/VIS, Shanghai, China). The quality of RNA was determined by 1% agarose gel electrophoresis.

### Identification of DEGs by ACP-based RT-PCR

Genes differentially expressed in bacteria in response to pBD2 treatment were identified using the GeneFishing^TM^ DEG Kit (Seegene, Seoul, South Korea) following the manufacturer’s instructions using 20 different arbitrary primers in combination with an oligo-dT anchor primer. Total RNAs extracted from the bacteria were converted into first-strand cDNAs using reverse transcription-PCR (RT-PCR) in 20 μl reaction containing 1 μl RNA, 4 μl of 5 × PrimeScriptII buffer, 1 μl of dNTP mixture (each 10 mM), 1 μl of 10 μM dT-ACP1, 0.5 μl of RNase inhibitor (40 U/μl), and 1 μl of PrimeScriptII RTase(200U/μL) at 42 °C for 1.5 h. First-strand cDNAs were stored at *−*20 °C until use for PCR. DEGs were screened with the ACP-PCR based method using primers that anneal specifically to the template in 20 μl of reaction containing 1 μl of cDNA, 1 μl of dT-ACP2 (10 μM), 1 μl of 10 μM arbitrary ACP, and 13 μl of premix. The PCR protocol for second-strand cDNA synthesis included one cycle at 94 °C for 5 min, followed by 50 °C for 3 min, and 72 °C for 1 min. The second-stage PCR amplification was then performed using 40 cycles of 94 °C for 40 sec, followed by 65 °C for 40 sec, 72 °C for 40 sec, and a 10 min final extension at 72 °C. The amplified PCR products were separated and analyzed on 2% agarose gels stained with ethidium bromide. The ACP-based RT-PCR was repeated three times. The differentially amplified PCR fragments were extracted from the gel using a DNA purification kit (TIANGEN, China) and directly cloned into a pMD18-T vector and transformed into *Escherichia coli* TG1 cell. The cloned fragments were subjected to DNA sequencing to characterize the identity of the DEGs.

### Analysis of the DEGs

All sequences were subjected to BLAST analysis using the bacterial genome database in GenBank and the Ensembl (http://bacteria.ensembl.org) genome browser to characterize the functional annotations.

### Quantitative real-time PCR

Reverse transcription was performed as described above. Differently expressed genes identified in ACP-RT-PCR were further confirmed by a quantitative real-time PCR (qRT-PCR) method performed as follows: 40 cycles of denaturation at 95 °C for 15 sec, annealing at 60 °C for 40 sec, and extension at 72 °C for 15 sec, according to the manufacturer’s protocol. Assays were performed with SYBR Green Dye (TaKaRa, Dalian, China) using a Bio-Rad CFX96 real-time PCR platform. All the samples were analyzed in triplicate and fold-changes of gene expression were calculated by 2^−△△CT^ methods with GAPDH as a reference gene.

### Statistical analysis

An analysis of variance (ANOVA) was performed with SPSS 17.0 software. LSD or Dunnett’s method was used to compare treatment means. Statistical significance was defined as *P* < 0.05.

## Electronic supplementary material

Supplementary Figure
