# Supplementary material for: Analysis of the antimicrobial mechanism of porcine beta defensin 2 against E. coli by electron microscopy and differentially expressed genes
Source: Sci Rep. 2018 Oct 2;8:14711. doi: 10.1038/s41598-018-32822-3 (PMC6168601; doi:10.1038/s41598-018-32822-3)
Supplement: Supplementary file 1 — Supplementary Figure [file 41598_2018_32822_MOESM1_ESM.pdf]

Analysis of antimicrobial mechanism of porcine beta defensin 2 against *E. coli* by electron microscopy and differentially expressed genes

Rui-bo Chen, Kun Zhang, Heng Zhang, Chun-yu Gao, Chun-li Li*

Department of Animal and Veterinary Science, Henan Agricultural University, Zhengzhou, 450002, Henan, The People's Republic of China

* *Corresponding author: Chun-li Li*

Address: Department of Animal and Veterinary Science, Henan Agricultural University, No. 15, Longzi Lake Campus, Zheng Dong New district, Zhengzhou, 450046, Henan Province, The People's Republic of China

Cell phone: + 86-15903997817

E-mail: hnclli@163.com

Supplementary figure

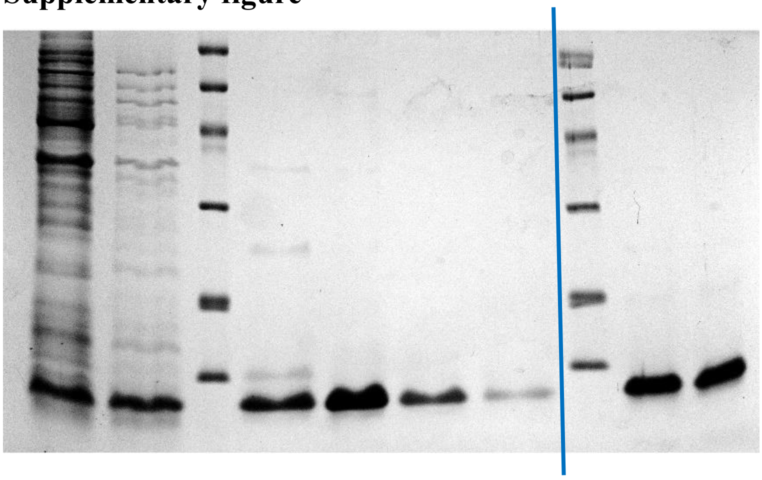

Figure 1 Analysis of expressed fusion and purified pBD2 and its antimicrobial activity (A) and (B) of figure 1 in the manuscript cropped from left part and right part of this figure respectively.

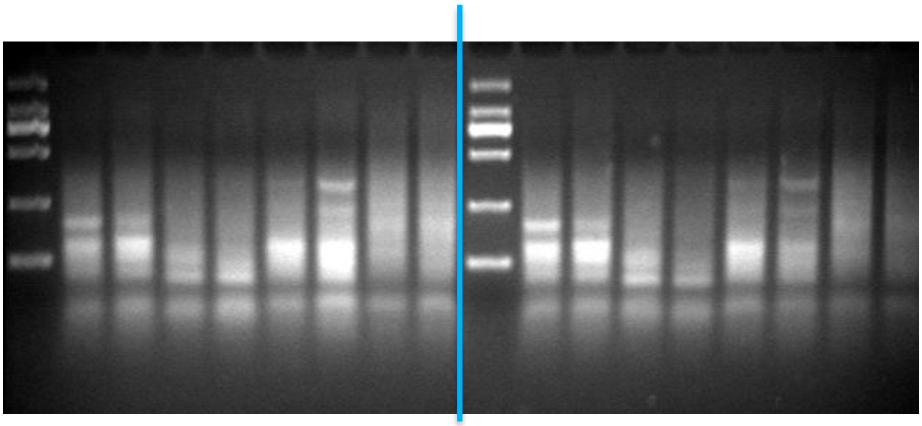

Figure 2 The identification of DEGs between control and pBD2-treated by ACP 1-4 at 37.5 μg/mL for 4 h.
The ACP 1-4 of figure 5 in the manuscript cropped from the left part of this figure.

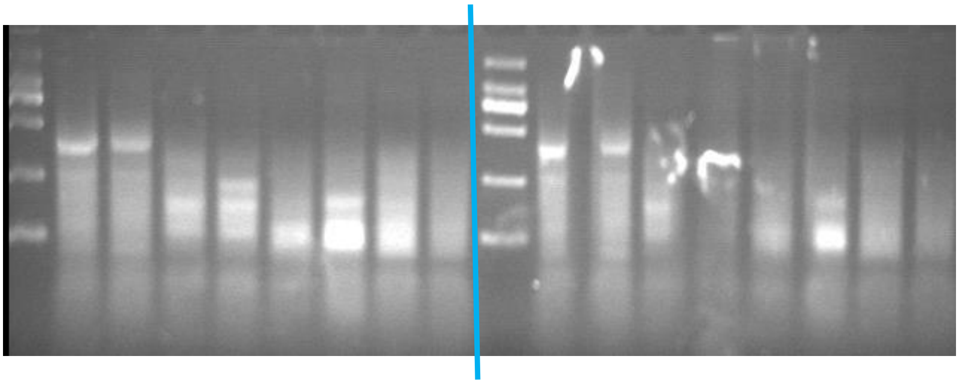

Figure 3 The identification of DEGs between control and pBD2-treated by ACP 5-8 at 37.5 μg/mL for 4 h.
The ACP 5-8 of figure 5 in the manuscript cropped from the left part of this figure.

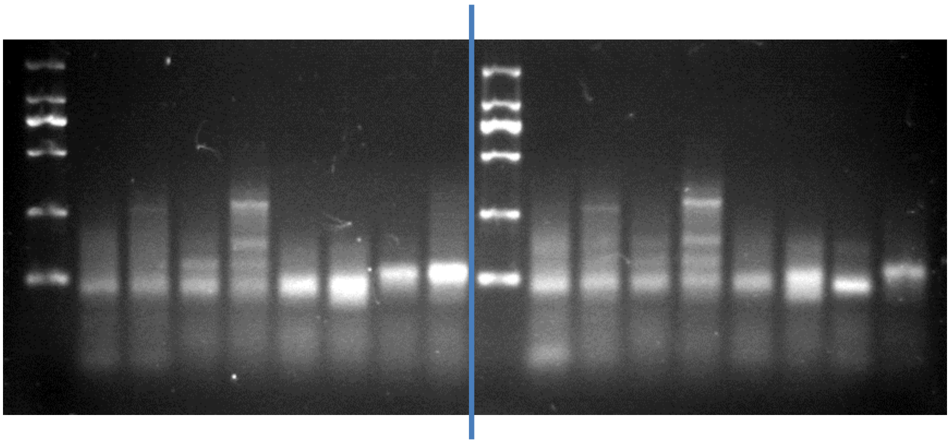

Figure 4 The identification of DEGs between control and pBD2-treated by ACP 9-12 at 150 μg/mL for 4 h.

The ACP 9-12 of figure 5 in the manuscript cropped from the right part of this figure.

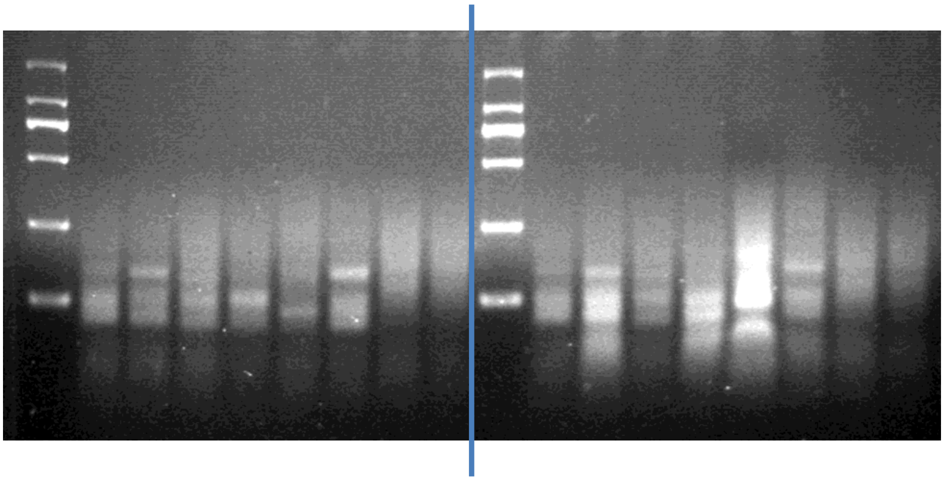

Figure 5 The identification of DEGs between control and pBD2-treated by ACP 13-16 at 150 μg/mL for 1h.

The ACP 13-16 of figure 5 in the manuscript cropped from the left part of this figure.

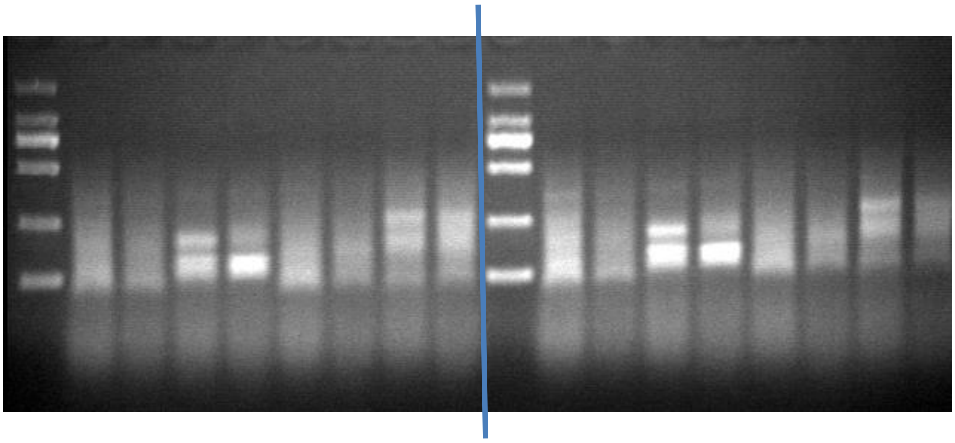

Figure 6 The identification of DEGs between control and pBD2-treated by ACP 17-20 at 150 μg/mL for 1h.

The ACP 17-20 of figure 5 (at the left) in the manuscript cropped from the right part of this figure.

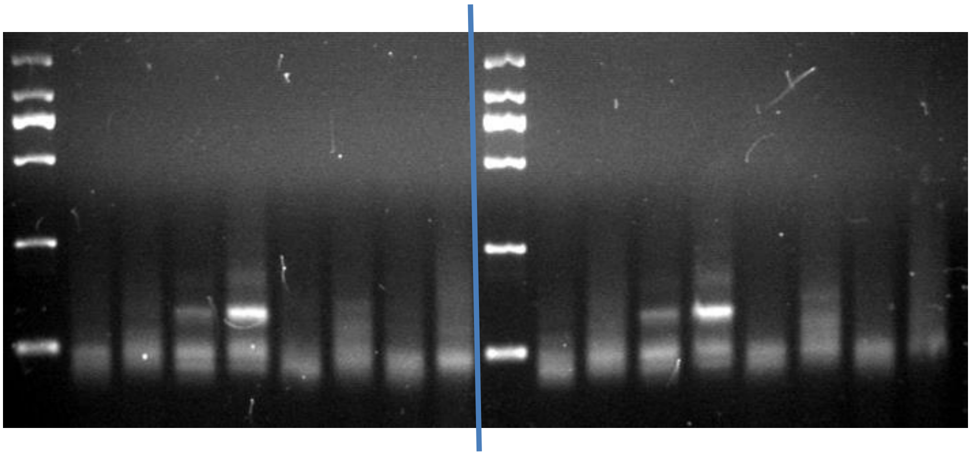

Figure 7 The identification of DEGs between control and pBD2-treated by ACP 17-20 at 150 μg/mL for 4 h.

The ACP 17-20 of figure 5 (at the right) in the manuscript cropped from the left part of this figure。
